# Fatal Stricture of the Colon

**Published:** 1830-04-01

**Authors:** 


### XXXI.

### Fatal Stricture of the Colon.

Organic diseases creep on, sometimes, with a pace as silent and fatal as that of the Indian Band, who, treading in each other's footsteps, give no indication of more than the approach of a solitary individual. So it is with a train of diseases, which are all masked by a single symptom, till the fatal termination approaches. The Editor of this Journal, was called in consultation to a lady in Lambeth, who had been ailing for several months, with torpor or constipation of the bowels, and without any other prominent symptom. The abdomen had been repeatedly examined by Dr. Stanton, but nothing could be detected to account for certain uneasy feelings in that region. Suddenly an aggravation of these feelings was experienced, and to the surprise of the medical attendant, a tumour was felt in the right hypochondriac region. Dr. Johnson was then called in, and attentively examined the abdomen of the patient. A roundish, but not hard swelling was felt in the region of the caput coli, and, on following the colon, all traces of this swelling disappeared till the middle of the transverse arch of the tube was gained, where another oblong, and densely hard swelling was felt, with a depression in its centre. Both of these swellings conveyed a sense of crepitation, on handling them. Dr. Stanton had come to the conclusion that these tumours were collections of fæculent matters in the colon, and, on mature consideration of all the circumstances, but more especially the sudden growth of them, Dr. J. became convinced that they could be nothing else than accumulations in the colon. On this supposition every means for unloading the bowels, by enemata and laxatives, were assiduously employed, but without the slightest advantage. Vomiting became the distressing symptom, and no substantial evacuation of the bowels could be procured. Sir Astley Cooper's assistance was put in requisition, but without any relief. For seventeen days the unfortunate lady lived, without any effectual passage through the bowels, and with daily vomiting. At length stercoraceous matters were thrown up—nothing could be retained in the stomach — emaciation made rapid progress—and death terminated the scene — the tumours remaining nearly the same as when first examined by Dr. Johnson. Difficulties almost insuperable were thrown in the way of a post mortem inspection. In the middle of the night, when the daughter was asleep, Dr. Stanton and Dr. Johnson proceeded to Lambeth, and examined the body. On opening the abdomen, the intestines were found universally adherent by inflammatory exudation, partly recent, partly of long standing. About the middle of the transverse arch of the colon, the coats of that intestine were half an inch in thickness, and the canal totally obliterated. The end of the little finger could not be forced through, without tearing the parts. From that point back to the valve of the colon, and even beyond the valve, the intestines were enormously distended with fæces. This was the sum total of the pathology—the fons, origo, et finis of the malady which caused the patient's death. The unfortunate lady had been in the habit, perhaps the necessity, of taking strong aperients, or rather purgatives, for years previously, and there can be little doubt that purgation aggravated the evil, when the stricture of colon was beginning to obstruct the passage of fæces along the narrowing tube. That life might have been preserved, many years, had a system of using enemata been rigidly and daily employed, we are fully convinced, for the liquefaction of the fæces by such measure would have taken off a great source of irritation, and facilitated the diurnal removal of all offending matters.

---

